# Repeated faecal microbiota transplantation for individuals with type 1 diabetes and gastroenteropathy

**DOI:** 10.1007/s00125-025-06544-x

**Published:** 2025-09-18

**Authors:** Katrine L. Høyer, Ditte S. Kornum, Simon M. D. Baunwall, Mette W. Klinge, Asbjørn M. Drewes, Knud B. Yderstræde, Susan Mikkelsen, Christian Erikstrup, Klaus Krogh, Christian L. Hvas

**Affiliations:** 1 Department of Hepatology and Gastroenterology, Aarhus University Hospital, Aarhus, Denmark; 2 Department of Clinical Medicine, Aarhus University, Aarhus, Denmark; 3 Steno Diabetes Center Aarhus, Aarhus University Hospital, Aarhus, Denmark; 4 Mech-Sense, Department of Gastroenterology and Hepatology, Aalborg University Hospital, Aalborg, Denmark; 5 Steno Diabetes Center North Denmark, Aalborg University Hospital, Aalborg, Denmark; 6 Steno Diabetes Center Odense, Odense University Hospital, Odense, Denmark; 7 Department of Clinical Immunology, Aarhus University Hospital, Aarhus, Denmark

**Keywords:** Autonomic neuropathy, Diabetic gastroenteropathy, Faecal microbiota transplantation

## Abstract

**Aims/hypothesis:**

Faecal microbiota transplantation (FMT) may alleviate gastrointestinal symptoms in individuals with diabetic gastroenteropathy, as demonstrated in a recent placebo-controlled trial. In most participants, symptom relief was transient, raising the need for repeated treatments. This study assessed the long-term efficacy, safety and feasibility of repeated, on-demand FMT as a maintenance treatment in this patient population.

**Methods:**

All 20 participants from the randomised clinical trial were offered extended open-label treatment with FMT. Symptom assessments were conducted by telephone every 2–3 months using the Gastrointestinal Symptom Rating Scale for Irritable Bowel Syndrome (GSRS-IBS). Secondary measures included bowel movement frequency, stool consistency assessed using the Bristol Stool Scale, perceived treatment benefit on a seven-point Likert scale, and adverse events (AEs). FMT was primarily given as oral capsules, and colonoscopy was used for participants who could not swallow capsules.

**Results:**

Of the original 20 participants, 17 were included in the present study and followed from September 2021 to December 2024, with a median Duration of follow-up of 33.2 months (range 14.7–39.1 months). Participants received a total of 95 FMT treatments, with a median of five per participant and a median interval of 5.3 months between treatments. FMT induced consistent symptom relief, with reduced GSRS-IBS scores across multiple treatments. At the last FMT treatment provided, the mean GSRS-IBS score had decreased from 60 (95% CI 54, 66) at baseline to 35 (95% CI 29, 40), with a mean difference of −25 (95% CI −18, −33). The occurrence of frequent bowel movements 2 weeks after treatment (> 7 per day) decreased from 19% (95% CI 10%, 28%) to 3% (95% CI 0%, 7%). Stool consistency improved after treatment, and the frequency of normal stool types (Bristol Stool Scale score 3–5) increased from 28% (95% CI 18%, 39%) to 76% (95% CI 66%, 86%). Participant satisfaction was high, with 86% reporting considerable benefits (Likert scores 5–7). Repeated FMT was generally well tolerated, with most AEs being mild and self-limiting. Fifteen serious AEs were documented, of which only one was deemed to be possibly related to FMT.

**Conclusions/interpretation:**

Repeated, on-demand FMT is effective and safe for long-term treatment of individuals with type 1 diabetes and severe diabetic gastroenteropathy.

**Trial registration:**

ClinicalTrials.gov NCT04749030

**Funding:**

The study was funded by a Steno Collaborative Grant (no. 0058906)

**Graphical Abstract:**

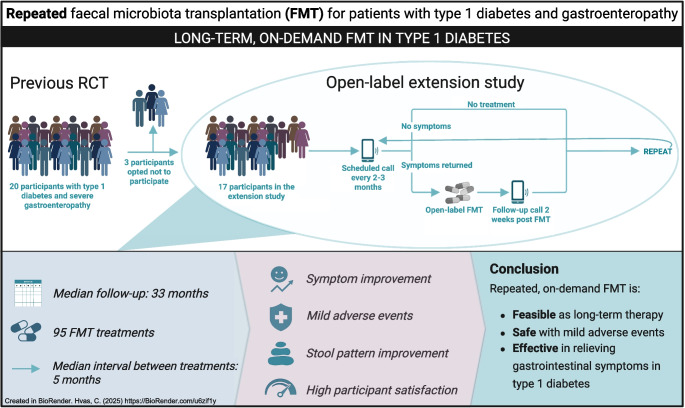


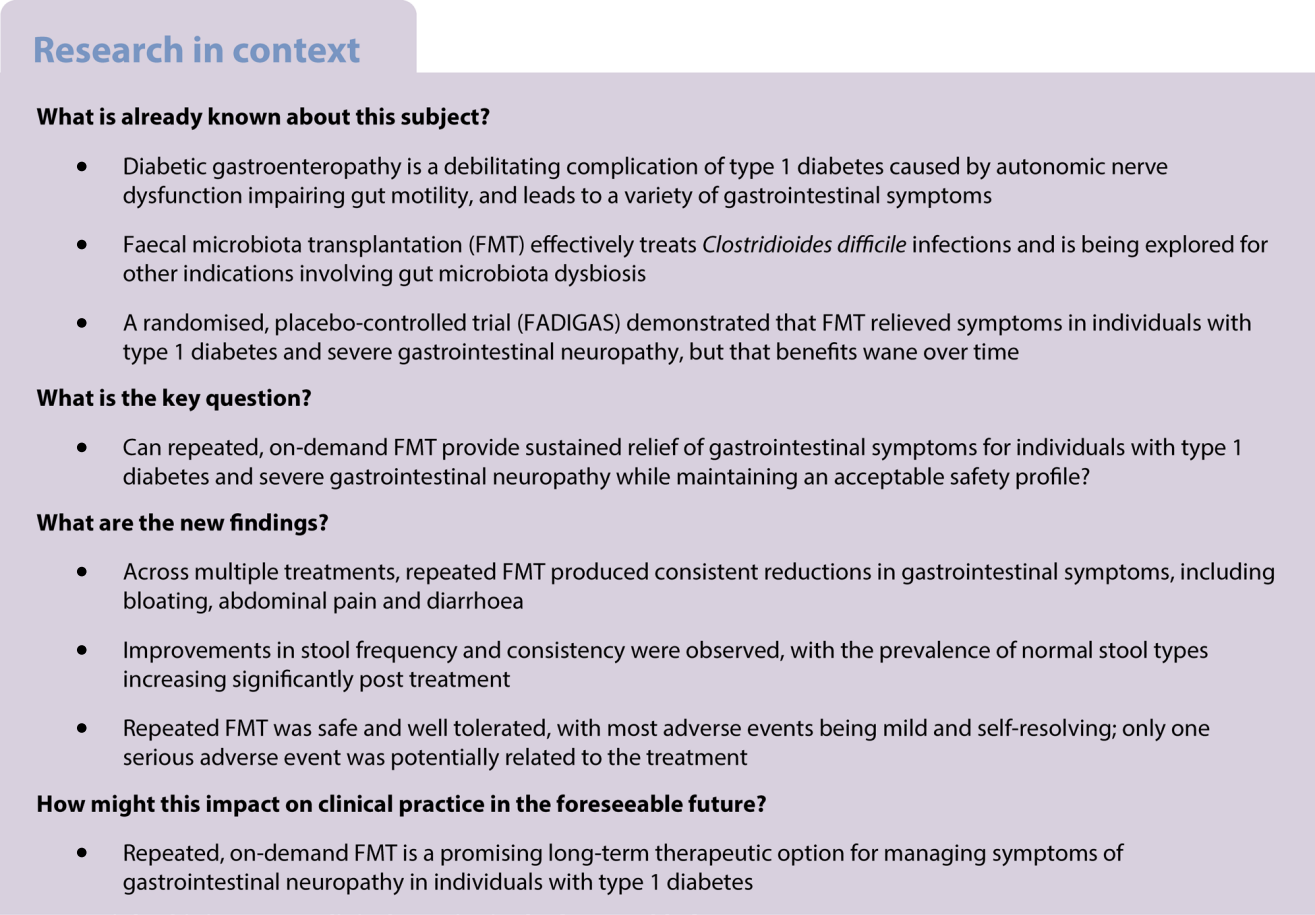


## Introduction

Diabetic gastroenteropathy is a debilitating complication of diabetes, often leading to chronic symptoms such as bloating, nausea, constipation and diarrhoea, considerably impairing quality of life [1]. Symptoms mainly result from autonomic neuropathy, which affects the enteric and autonomic nervous system, disrupting normal gut motility, secretion and absorption [2, 3]. The nerve damage is considered irreversible, and treatment strategies aim to halt progression and treat symptoms rather than provide a cure [4]. Symptomatic management remains a clinical challenge, given the limited therapeutic options and a high risk of severe side effects [5, 6].

The gut microbiota has emerged as a critical modulator of gastrointestinal health, affecting gut motility, immune function and epithelial barrier integrity [7]. Gut microbiota alterations, or dysbiosis, are thought to be implicated in the pathogenesis of conditions associated with gut dysmotility and inflammation [8]. This has sparked increased interest in gut microbiota-based therapies, particularly faecal microbiota transplantation (FMT), which involves the transfer of gut microbiota from healthy donors to patients. FMT has shown consistent efficacy in recurrent *Clostridioides difficile* infection [9], but its use in other conditions, such as inflammatory bowel disease or irritable bowel syndrome, has yielded conflicting results [10]. Some studies have reported symptom relief, while others found limited or no benefit, reflecting the gut microbiota’s complex and variable role in gastrointestinal disorders. The reasons for the outcome variability are probably multifactorial, influenced by differences in disease pathophysiology, patient populations, donor selection and study protocols [11, 12]. Furthermore, the gut microbiota is a dynamic ecosystem that may vary significantly between individuals, complicating treatment strategies [13].

The FADIGAS safety and pilot efficacy study demonstrated that FMT provided significant symptom relief and improved quality of life in individuals with type 1 diabetes and severe gastrointestinal symptoms [14]. Symptom relief was temporary, with symptoms gradually recurring over time, necessitating repeated treatments. This suggests that individuals with severe gastrointestinal neuropathy may be predisposed to a fundamental and recurring microbial dysbiosis, probably related to their intestinal environment, including changes in motility and transit times [15, 16].

In the present open-label extension study, we investigated the long-term use of FMT. FMT was offered on demand, based on an individual’s symptomatic presentation. We hypothesised that repeated FMT treatments would provide sustained symptom relief. Thus we assessed the clinical use of repeated, on-demand FMT and its potential to provide long-term relief in a real-world clinical setting while monitoring safety and gastrointestinal symptoms.

## Methods

### Study design and population

This open-label extension study enrolled individuals who had completed the primary FADIGAS study. They were invited to participate in the extended study, participating in regular follow-up telephone assessments and receiving FMT on demand [14]. The FADIGAS study was an investigator-initiated, double-blinded, placebo-controlled clinical trial conducted at Aarhus University Hospital, Denmark, to evaluate the safety and effects of FMT in individuals with type 1 diabetes and severe gastroenteropathy symptoms. Ethical approval for the extension was covered by the original protocol, approved by the Central Denmark Region Committee on Health Research Ethics (j.no. 1-10-72-345-20). Participation in the open-label phase was voluntary and based on the informed consent obtained at enrolment in the FADIGAS trial.

The source population consisted of adults with type 1 diabetes and severe gastrointestinal symptoms, recruited from outpatient clinics in the Central Denmark Region. The study sample reflects this population in terms of age and sex distribution. Sex was not used as an inclusion or exclusion criterion, and the study was not specifically designed to compare outcomes by sex or gender. Ethnicity data were not collected, as the study was conducted in a population that is ethnically very homogeneous; all trial participants were of European descent. As the study was conducted within a publicly funded healthcare system with equal access, the sample is considered broadly representative with respect to regional and socioeconomic factors.

In the FADIGAS study, eligible individuals had had type 1 diabetes for over 5 years and a score on the Gastrointestinal Symptom Rating Scale for Irritable Bowel Syndrome (GSRS-IBS) of 40 or above. The sex of participants was recorded based on self-report at enrolment. Key exclusion criteria included severe renal insufficiency, active *C. difficile* infection, chronic gastrointestinal diseases, recent antibiotic use and prior major abdominal Surgery. Participants were initially randomised to receive FMT or placebo under blinded conditions before entering an open-label phase During which they all had access to FMT. The extension study was initiated in September 2021, after completion of the primary study. Data were extracted from the primary study database in December 2024.

### Follow-up protocol

**Fig. 1:**
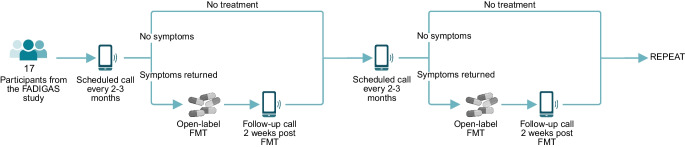
The extension study included follow-up calls every 2–3 months to assess gastrointestinal symptoms. Participants with recurring symptoms received additional open-label FMT, while symptom-free participants continued regular follow-up calls. Two weeks after each FMT, symptom scores were reassessed, a procedure that was introduced 1 year into the study. Created in BioRender. Hvas, C. (2025) https://BioRender.com/xtq0qi9

The study involved scheduled follow-up telephone calls from an investigator every 2–3 months to assess gastrointestinal symptoms using the GSRS-IBS questionnaire (Fig. 1). Participants who experienced gastrointestinal symptom recurrence were offered additional open-label FMT treatment. Eligibility for retreatment was based on a GSRS-IBS total score exceeding 40, indicating moderate-to-severe symptoms, in combination with participant-reported symptom recurrence and clinician assessment. A minimum interval of 2 months was required between FMT treatments to prevent over-treatment. Those who remained symptom-free were scheduled for a follow-up telephone call after a further 2–3 months. Participants were also encouraged to contact the department if symptoms recurred. From September 2022—approximately 1 year after initiation of the open-label extension—a follow-up telephone consultation was introduced 2 weeks after FMT, during which GSRS-IBS symptom scores were recorded. By this time, some participants had already received several additional FMTs as part of the extension protocol.

In addition to gastrointestinal symptoms, participants were invited to report their daily bowel movement frequency and stool consistency, with the latter based on the Bristol Stool Scale [17]. These reports were collected the week preceding each scheduled call and at the 2-week post-treatment follow-up assessment. At the post-treatment assessment, participants assessed their perception of treatment efficacy using a 7-point Likert scale, focusing on symptom relief [18]. They specifically assessed the statement ‘The FMT treatment helped my gastrointestinal symptoms’, with 7 representing ‘completely true’ and 1 representing ‘completely untrue’. Any adverse events (AEs) occurring during or after FMT treatments were reported.

### Symptom scores

The score for the GSRS-IBS, a validated tool developed to assess gastrointestinal symptoms [19], served as the primary outcome measure. It includes items that address key symptom domains Such as abdominal pain, bloating, diarrhoea and constipation, allowing comprehensive evaluation of symptom severity. Scores are aggregated into a total score, with higher scores indicating greater symptom severity. The total score represents the Sum of all scores. Subscores were calculated by summing scores from the relevant questions for each specific subcategory and dividing the total by the number of questions in that subcategory. GSRS-IBS questionnaires were collected at the scheduled visit before administering FMT and again 2 weeks after treatment. Only participants with complete pre-treatment and post-treatment scores were included in the data analysis.

### FMT procedure

FMT was administered according to established protocols from the FADIGAS study [14]. The primary method of administration was oral capsules [20], which are favoured for their non-invasive nature and patient convenience. For one participant who was unable to swallow capsules, FMT was delivered via colonoscopy [21, 22]. Donors were rigorously screened according to international guidelines to ensure safety and quality [23]. At each FMT, participants received faeces from a single donor, with randomised donor allocation to ensure unbiased pairing of donors and recipients. Throughout the multiple FMT treatments, participants were exposed to microbiota from different donors. If a participant experienced minimal or no symptom relief after an FMT and required a subsequent treatment, a different donor was selected to optimise therapeutic efficacy.

### Long-term AE follow-up

The safety and tolerability of repeated FMT were assessed through participant-reported outcomes and clinical monitoring. AEs were systematically classified and graded using the Common Terminology Criteria for Adverse Events (CTCAE), version 5.0 [24]. These criteria categorise events into specific terms, and assign severity grades from 1 (mild) to 5 (death) based on their impact on patient well-being and the level of medical intervention required.

### Statistical analyses

Data were collected using REDCap (https://redcap.au.dk). Data distribution was evaluated by inspecting QQ plots and histograms. For data with distributions that met the assumptions for parametric analysis, the results are expressed as mean values, accompanied by 95% CI, and analysed using paired *t* tests. Medians and ranges are reported for data that did not meet these assumptions, and non-parametric tests were applied. Where applicable, frequency counts are provided with corresponding percentages. GSRS-IBS scores were analysed using a linear mixed-effects model, with time point as a fixed effect and participant ID as a random effect. Due to the small sample size, the Kenward–Roger method was used to estimate degrees of freedom. Model assumptions were evaluated using diagnostic plots of residuals, fitted values and best linear unbiased predictions for the random effects. The estimated mean difference in GSRS-IBS score from the start of the study until after the last FMT is reported with 95% CIs and corresponding *p* values. Statistical significance was set at *p*<0.05. All statistical analyses were performed using Stata version 17.0 (StataCorp, College Station, TX, USA).

## Results

### Extension study participation

Seventeen (85%) of the 20 participants included in the FADIGAS study wished to continue in the open-label extension study. Of the three participants who opted not to participate, one had undergone a beta cell transplant and was on immunosuppressive therapy. Two had noticed no relevant symptom improvement.

**Fig. 2:**
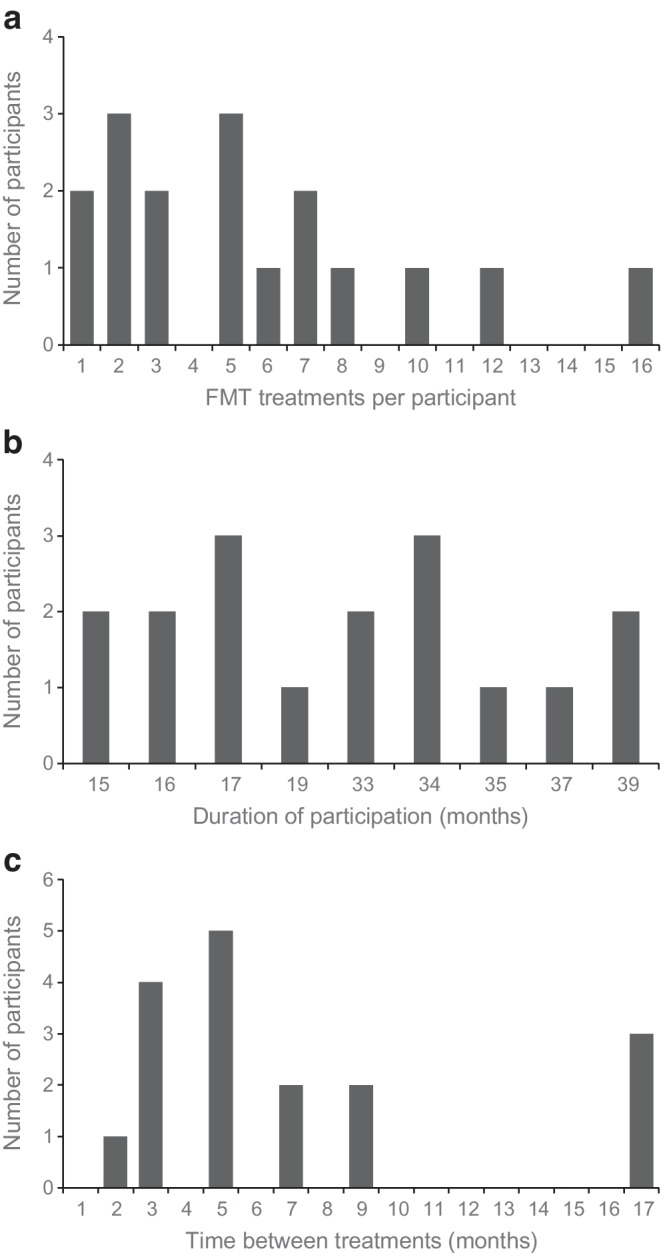
Overview of FMT treatment characteristics and duration of participation in the open-label study. (**a**) Distribution of number of FMT treatments; (**b**) distribution of duration of participation in the open-label study from enrolment to the study cut-off date; (**c**) distribution of interval between FMT treatments

After completing the randomised study, participants were contacted for the first time after a median of 63 days (IQR 42–77), at which time 13 of the 17 participants expressed a wish for additional FMT treatments due to symptom recurrence. The remaining four participants were experiencing sustained symptom relief at this time but later had symptom recurrence, prompting treatment. Distribution of number of treatments, duration of participation and treatment intervals are illustrated in Fig. 2*.*

### Clinical outcomes

**Fig. 3:**
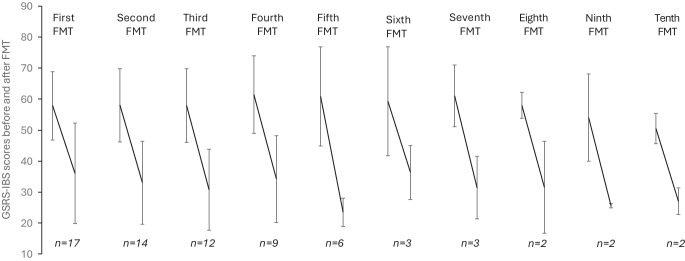
GSRS-IBS scores before and after open-label treatment with FMT, including only treatments for which data were available both before and after the intervention. Each pair of data points represents a new FMT treatment cycle. For each pair, the first data point corresponds to the mean GSRS-IBS score before the initial FMT, and the second point reflects the mean score after that treatment for all eligible participants at each time point. Error bars indicate the SD, illustrating score variability

In all 17 participants, FMT significantly reduced gastrointestinal symptoms across multiple treatment rounds, as measured by the GSRS-IBS total score and its subcategories, including pain, bloating, constipation, diarrhoea and early satiety (Fig. 3). Participants received a median of five treatments (range 1–17) During a median follow-up Duration of 33.2 months (range 14.7–39.1 months).

**Fig. 4:**
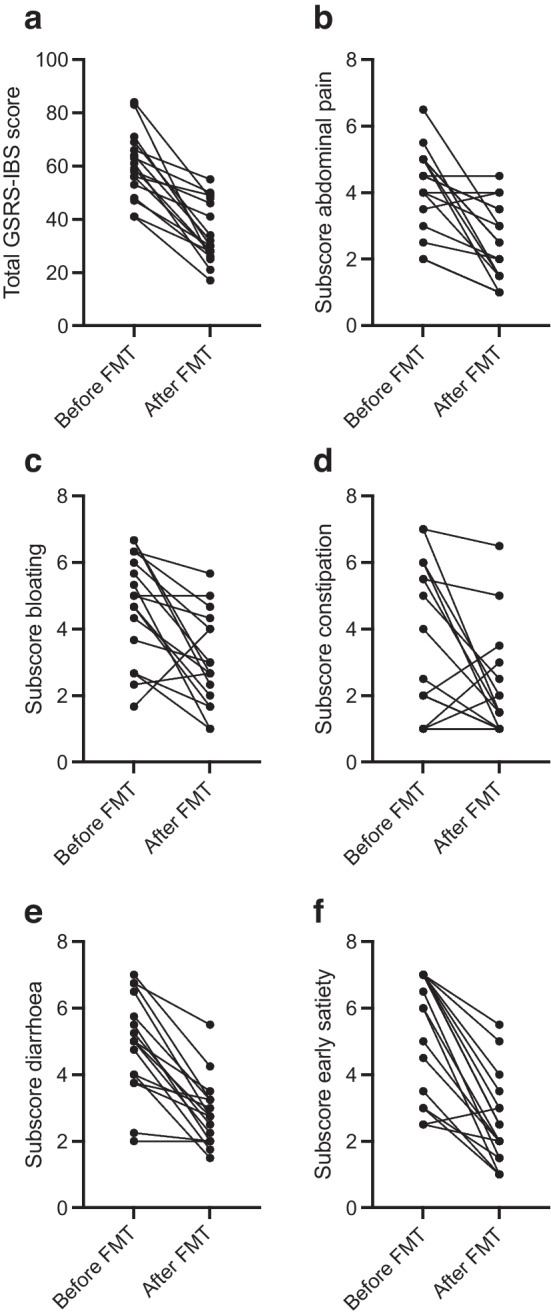
Mean GSRS-IBS total score (**a**) and subscores for abdominal pain (**b**), bloating (**c**), constipation (**d**), diarrhoea (**e**) and early satiety (**f**) for all 17 participants, before treatment and after the final FMT they received prior to data extraction. Each line represents the change in symptom severity for an individual participant. Decreases in symptom scores indicate improvement. Fewer than 17 lines are visible in some plots because lines from different participants overlap when identical or near-identical scores were reported

The mean GSRS-IBS score after each FMT decreased from 60 (95% CI 54, 66) before treatment to 35 (95% CI 29, 40), based on the score recorded at the time of the last FMT administered prior to data extraction, corresponding to a mean difference of −25 (95% CI −18, −33) (Table 1). The diarrhoea Subscore decreased from 4.9 (95% CI 4.1, 5.6) to 2.8 (95% CI 2.3, 3.4), corresponding to a mean difference of −2.0 (95% CI −1.4, −2.6). The early satiety Subscore decreased from 5.4 (95% CI 4.5, 6.3) to 2.5 (95% CI 1.8, 3.2), corresponding to a mean difference of −2.9 (95% CI −2.0, −3.7). Constipation Subscores improved from 3.5 (95% CI 2.3, 4.7) to 2.1 (95% CI 1.3, 2.9), with a mean difference of −1.4 (95% CI −0.2, −2.5). Pain and bloating similarly decreased substantially (Table 1). Individual changes before and after the final treatment are illustrated in Fig. 4, showing consistent symptom reduction across participants. A linear mixed-effects model confirmed a statistically significant GSRS-IBS score reduction following FMT compared with pre-treatment (mean reduction 25.7 points; 95% CI 22.7, 28.6; *p*<0.0001), supporting the consistency of the observed symptom relief across repeated treatments.

**Table 1:** Changes in symptom scores following repeated FMT in individuals with diabetic gastroenteropathy

Symptom	Before FMT	After FMT	Mean difference
GSRS-IBS score	60 (54, 66)	35 (29, 40)	−25 (−18, −33)
Abdominal pain	4.2 (3.5, 4.8)	2.5 (1.9, 3.1)	−1.7 (−0.9, −2.4)
Bloating	4.7 (3.9, 5.5)	3.0 (2.3, 3.7)	−1.7 (−0.8, −2.6)
Constipation	3.5 (2.3, 4.7)	2.1 (1.3, 2.9)	−1.4 (−0.2, −2.5)
Diarrhoea	4.9 (4.1, 5.6)	2.8 (2.3, 3.4)	−2.0 (−1.4, −2.6)
Early satiety	5.4 (4.5, 6.3)	2.5 (1.8, 3.2)	−2.9 (−2.0, −3.7)

Symptoms were assessed using the GSRS-IBS total score and its Subcategories. Mean scores before and after treatment are shown, together with 95% CIs. Mean differences between scores before and after treatment are also provided with the corresponding 95% CIs

### Stool frequency and consistency

The differences in stool frequency and consistency between the week preceding the scheduled follow-up call and 2 weeks after the FMT treatment were available for 72 treatment rounds, with a median of four treatments per participant and a range of 1–12 (Table 2).

**Table 2:** Stool frequency and Bristol Stool Scale results before and after FMT

	Before FMT	After FMT
Stool frequency		
0	7 (10)	3 (4)
1	19 (26)	37 (51)
2	3 (4)	18 (25)
3	10 (14)	1 (1)
4	5 (7)	6 (8)
5	6 (8)	2 (3)
6	5 (7)	1 (1)
7	3 (4)	2 (3)
> 7	14 (19)	2 (3)
Bristol Stool Scale score		
1	3 (4)	2 (3)
2	11 (15)	2 (3)
3	7 (10)	12 (17)
4	6 (8)	28 (39)
5	7 (10)	15 (21)
6	28 (39)	9 (13)
7	10 (14)	4 (6)

Values are *n* (%) for 72 treatment rounds

Stool frequency before treatment was between zero and three stools per day in 54% of participants (95% CI 43, 66%), while stool frequencies of between four and seven stools per day were reported by 26% (95% CI 16, 36%). Stool frequencies of more than seven stools per day were reported in 19% of participants (95% CI 10, 28%). Two weeks after treatment, 82% of participants (95% CI 73, 91%) reported between zero and three stools per day. Frequencies between four and seven stools per day were reported by 15% of participants (95% CI 7, 23%), and only 3% of participants (95% CI 0, 7%) reported stool frequencies exceeding seven stools per day. The mean stool frequency was reduced from 4.6 stools per day (95% CI 3.5, 5.7) to 2.1 (95% CI 1.6, 2.5) after FMT (*p*<0.0001).

Stool consistency, assessed using the Bristol Stool Scale, changed markedly after FMT. Before FMT, hard stools (types 1 and 2) were reported in 19% of participants (95% CI 11%, 29%), whereas liquid stool (types 6 and 7) accounted for 53% of participants (95% CI 41%, 65%). Two weeks after FMT, the prevalence of hard stools was reported by 5.6%, i.e. a 72% reduction (95% CI 1%, –12%), whereas liquid stools decreased by 66% to 18% (95% CI 10%, 27%).

### Participant satisfaction

Participant satisfaction with FMT was assessed using a 7-point Likert scale. At the 2-week follow-up assessment after FMT (*n*=72), the mean satisfaction score was 5.8 (95% CI 5.4, 6.1). A total of 86% of responses fell within the positive range of 5–7, with 44% selecting 7 (‘completely true’), 25% selecting 6 (‘mostly true’) and 17% selecting 5 (‘somewhat true’). Lower ratings were less common, with 6% selecting 4 (‘uncertain’) and 8% selecting 1 (‘completely untrue’) (Table 3).

**Table 3:** Participant satisfaction with FMT at the 2-week follow-up assessments conducted after each FMT administration (*n*=72) based on a 7-point Likert scale

Score	Two-week follow-up assessment (*n*=72)
7 (completely true)	32 (44)
6 (mostly true)	18 (25)
5 (somewhat true)	12 (17)
4 (uncertain)	4 (6)
3 (somewhat untrue)	0 (0)
2 (mostly untrue)	0 (0)
1 (completely untrue)	6 (8)

Values are *n* (%)

Scores range from 7 (‘completely true’) to 1 (‘completely untrue’), with higher scores (5–7) indicating satisfaction, and 4 representing a neutral response

### Safety and tolerability

At the 2-week follow-up assessment (safety was assessed in 69 of the 72 follow-up visits), participants were systematically asked about treatment-related AEs. AEs were reported at 28 follow-up assessments (41%) (Table 4). A total of 98 AEs were documented at the 2-week follow-up assessment or at other participant contacts throughout the study (Table 3). Most AEs were classified as mild (grade 1 according to CTCAE version 5). Moderate or severe AEs (grade ≥2 grade according to CTCAE version 5) accounted for 23 events, of which 14 were unrelated to FMT, and nine were deemed to be possibly related. Importantly, no AEs led to death, and most events resolved or improved during follow-up. The most frequently reported AEs were nausea, diarrhoea and abdominal pain. AEs did not increase with successive treatments, and events were evenly distributed across treatments, suggesting no cumulative risk with repeated FMT.

**Table 4:** AEs during the extension study

Event	Number
All AEs during the extension study	
Total number reported	98
Grade 1 (CTCAE version 5)	75
Grade ≥2 (CTCAE version 5)	23
AE leading to death	0
Resolved or improved	79
Resolved with sequelae	12
Not resolved during follow-up or unknown course	7
Occurred during administration	4
Occurred During follow-up within 24 h	51
Occurred after 24 h	43
Type of AE	
Nausea	21
Diarrhoea	15
Abdominal pain	9
Vomiting	7
Fatigue/malaise	7
Stomach rumbling	7
Bloating	5
Flushing	3
Dizziness	2
Reduced appetite	1
Constipation	1
Chills	1
Other	19
Details for AEs grade ≥2	
Not related to FMT	14
Possibly related to FMT	9
Diarrhoea	5
Abdominal pain	2
Nausea	1
Other	1

Only one serious AE was deemed to be possibly related to FMT. This participant presented with diarrhoea and vomiting and was hospitalised 9 days after FMT. The participant was a kidney transplant recipient, had preserved renal function at inclusion and did not meet any exclusion criteria. During the hospital stay, he received rehydration therapy and was treated for a urinary tract infection. No alternative causes for vomiting were identified and, while the event was conservatively classified as possibly related to FMT, no firm causal link was established. The participant recovered, was discharged without sequelae, and continued participation in the study without relapse of symptoms.

## Discussion

This open-label extension study demonstrated that repeated FMT is safe and effectively alleviates moderate-to-severe gastrointestinal symptoms in individuals with diabetic gastroenteropathy. Symptoms tended to recur over time, underscoring the need for repeated treatments to maintain symptom relief. The findings indicate that FMT may serve as a viable long-term therapeutic approach for managing severe gastrointestinal symptoms in individuals with type 1 diabetes.

### Mechanistic considerations

Diabetic gastroenteropathy disrupts gut motility through structural and functional changes, including neuropathy, smooth muscle atrophy and altered gut–brain axis communication [25, 26]. These changes may cause prolonged gastrointestinal transit times, favouring a dysbiotic microbial composition and exacerbating symptoms such as bloating, abdominal pain, diarrhoea and constipation [27]. Given the interplay between microbial dysbiosis and disease pathology, restoring a diverse and functionally balanced microbiota through FMT may help re-establish key microbial functions, including the production of short-chain fatty acids and regulation of gut inflammation [28, 29]. This may, in turn, lead to symptom relief. However, the chronic nature of the underlying neuropathy means that the dysbiosis-predisposing factors remain unchanged. This may explain why repeated treatments are required to maintain symptom relief.

The use of multiple donors over time was a pragmatic feature of this study, reflecting the clinical setting in which sustained donor consistency is rarely feasible. While donor variability is increasingly recognised as a factor influencing FMT efficacy, a clear understanding of what constitutes an effective donor is still lacking. No specific microbial or functional traits have yet been established to define donor ‘quality’. In light of this, we chose not to use mixed-donor preparations within individual treatments, in order to preserve donor identity and allow clearer interpretation of clinical outcomes. To preserve donor traceability and enable retrospective analyses of treatment responses, we deliberately used single-donor preparations rather than pooled material. This approach allows assessment of potential donor effects based on clinical outcomes alone, without requiring microbiota sequencing at this stage. Moreover, mixing donor samples would compromise future mechanistic insight by obscuring donor-specific contributions, and current evidence does not support increased efficacy when using pooled donors in indications such as irritable bowel syndrome [30]. In addition, from a regulatory standpoint, the use of pooled donor material conflicts with EU regulations for processing of substances of human origin, which require traceability and donor-specific documentation.

Individuals with type 1 diabetes exhibit distinct alterations in gut microbiota composition compared with healthy individuals [31]. Several studies have reported reduced microbial diversity in type 1 diabetes and depletion of beneficial short-chain fatty acid-producing bacteria, including *Faecalibacterium prausnitzii*, *Akkermansia muciniphila* and *Bifidobacterium* species [32, 33]*.* These bacteria are crucial in maintaining gut homeostasis by contributing to epithelial barrier integrity, modulating immune responses and supporting gut–brain signalling. Additionally, an over-representation of proinflammatory bacteria, including certain *Bacteroides* and Proteobacteria species, has been observed in individuals with type 1 diabetes [34].

Further research is necessary to increase our understanding of the specific microbial changes associated with symptom relief. Identifying predictive biomarkers of FMT responsiveness may potentially optimise donor selection and enhance individualised treatment strategies. Adjunctive therapies, such as dietary modifications or prebiotics, may help stabilise microbiota composition and prolong the effects of FMT. Future studies should explore these approaches to refine FMT protocols and improve long-term clinical outcomes in individuals with diabetic gastroenteropathy.

### Clinical efficacy

The overall GSRS-IBS score improved consistently after each FMT. This scale is widely used in clinical studies to evaluate the impact of interventions on gastrointestinal symptoms. Stool frequency improved after FMT, as shown by the substantial reduction in the percentage of participants reporting high stool frequencies (seven or more stools per day) from 19% at scheduled follow-up assessments to 3% at 2 weeks after treatment. Similarly, stool consistency improved, with the prevalence of normal stool types (Bristol Stool Scale scores 3–5) increasing from 28% During scheduled follow-up assessments to 76% at 2 weeks after treatment. Participant satisfaction scores reflected these clinical improvements, with 86% of responses indicating symptomatic relief.

The use of multiple donors over time reflects the practical realities of clinical FMT implementation, where long-term donor consistency may not be possible. This approach may have supported the sustained symptom relief observed in some participants, although this was not evaluated in the present study.

Symptom recurrence and the need for repeated treatments highlight the importance of understanding the underlying mechanisms of diabetic gastroenteropathy and optimising FMT protocols to achieve a sustained effect. Despite long-term benefits when applying multiple treatments, our findings do not indicate that FMT changes the underlying pathophysiology causing diabetic gastroenteropathy. The GSRS-IBS scores improved to levels comparable to those observed after the initial treatment in the randomised study, but before each subsequent treatment, the baseline scores had returned to levels similar to those recorded before the participants entered the randomised study. This suggests that while FMT provides effective symptom relief, the underlying condition remains chronic.

To Support interpretation of the observed effects in this extension study, we compared symptom scores with those from the preceding randomised trial. In that trial, the mean GSRS-IBS score decreased from 64 at baseline to 56 following placebo treatment and from 58 to 35 following FMT treatment (*p*=0.01). In the present study, the mean baseline score was 60, improving to 35 after the final FMT. While these findings must be interpreted with caution due to the absence of a comparator group, the magnitude and consistency of symptom relief across multiple treatments Suggest an effect that extends beyond natural symptom fluctuation or placebo responses. Notably, two of the 20 participants from the original trial declined to participate in the extension study Due to lack of symptomatic improvement, indicating that approximately 10% of participants did not experience meaningful clinical benefit. This highlights the importance of identifying predictors of treatment responses, and underscores the possibility that FMT may not be effective for all individuals with diabetic gastroenteropathy. No formal definition of response was applied in the present study, but our findings point to the need for standardised and validated response criteria and suggest possible elements of such criteria, including patient-reported global symptom improvement.

### Safety of repeated FMT

Use of FMT has never been reported as a long-term treatment option in any patient population. Therefore, strict attention to safety was pivotal. AEs were reported in 41% of participants at one or more follow-up assessments. Whereas most AEs were classified as mild and manageable, this finding emphasises the importance of strict AE monitoring and management. The predominance of mild AEs suggests that repeated FMT is generally well tolerated in this patient population. However, the occurrence of moderate or severe events indicates the need for careful clinical monitoring, especially for individuals with complex comorbidities [35]. The occurrence of a serious AE involving hospitalisation due to diarrhoea and vomiting illustrates the importance of identifying and mitigating risk factors that could predispose to severe complications, although it remains unclear whether FMT did in fact cause these symptoms.

Our findings align with those of previous studies that reported FMT to be a generally safe treatment. They also reinforce the need to tailor treatment protocols to individual patient profiles and ensure access to appropriate follow-up [36, 37]. Safety monitoring and reporting are necessary for optimising the long-term use of FMT and for establishing a comprehensive understanding of its risk–benefit profile in diverse clinical populations. The lower frequency of reported AEs in the extension study than in the placebo-controlled study may reflect the participants’ increased familiarity with FMT.

### Clinical implications

Our study has important clinical implications. Because of the irreversible nature of nerve damage, long-term symptom management remains a significant challenge for individuals with type 1 diabetes and severe gastrointestinal neuropathy. Repeated FMT may serve as a viable therapeutic option, providing individuals with symptom relief for a condition that has limited treatment options. By tailoring FMT administration to patient-reported symptom recurrence, clinicians can adopt a personalised approach that enhances treatment benefits and reduces unnecessary interventions.

Our findings highlight the potential of exploring FMT as a therapeutic strategy in other patient populations with similar symptom profiles or underlying pathophysiology. The pathophysiology behind bowel symptoms in type 2 diabetes shares features with that in type 1 diabetes [36, 38]. Future studies should evaluate the efficacy and safety of FMT in type 2 diabetes, assessing its potential to improve glycaemic management, reduce gastrointestinal symptoms and affect underlying inflammatory pathways. This may potentially extend the clinical use of FMT to a larger patient population.

### Limitations

Although the findings are extremely promising, important limitations should be considered. First, the lack of a placebo group in the extension study limits our ability to attribute symptom relief exclusively to FMT. However, the consistent improvements in GSRS-IBS scores and other endpoints support a causal effect of FMT. A placebo response was observed in the primary FADIGAS study, but it was much more limited than the effects recorded after FMT administration. This limited placebo effect nonetheless indicates that psychological factors and participant expectations may have contributed to symptom perception. However, the more pronounced improvement after FMT suggests that the treatment was the primary driver of the observed benefits. Future placebo-controlled, double-blinded studies are essential for further validation of FMT efficacy.

Second, the reliance on self-reported outcomes introduces a risk of subjectivity and recall bias. Although patient-reported outcome measures remain of crucial importance, integrating objective measures of gastrointestinal function, such as motility assessments or inflammatory biomarkers, may provide more robust evidence in future studies.

Third, because we did not include microbiota data in the present analysis, symptom relief could not be directly linked to specific microbial changes induced by FMT. Stool samples were collected during the study and are currently stored for future microbiome analyses. Such analyses should take into account donor variability, recipient characteristics and potential donor–recipient interactions in order to better understand their possible role in treatment response.

Finally, although 17 of 20 participants from the original study continued in the present study, the study population was relatively small, requiring more extensive studies to confirm our findings and identify predictors of treatment response. The participants had severe gastrointestinal symptoms, which may limit how broadly the findings can be applied to individuals with milder symptoms. In the present study, symptom assessments were conducted 2 weeks after each FMT. While this time point ensures standardised and timely reporting, it may not fully capture the Durability of the response. However, in our prior study comparable improvements were observed 4 weeks after FMT, supporting the relevance of measuring short-term outcomes [14]. In addition, a formal sex-based analysis was not performed due to the small sample size and the exploratory nature of the study. We acknowledge that this limits the ability to draw conclusions about potential sex-related differences in treatment response, and this should be considered when interpreting the findings.

### Future directions

In light of the observed pattern of symptom recurrence, we do not consider FMT to be suitable as a single administration intervention, but rather as a potential maintenance therapy in individuals with diabetic gastroenteropathy. Scheduled proactive FMT administration at fixed intervals, rather than symptom-driven retreatment, could help sustain more consistent symptom control. Alternative delivery strategies, such as administering lower daily doses via oral capsules at home, could provide a more patient-friendly and flexible approach. Monitoring stool microbial diversity may allow the timing of therapy to be refined, allowing earlier intervention before clinical relapse. Efforts to characterise key features of ‘effective donors’ may help enhance treatment consistency.

Use of adjunctive therapies, such as prebiotics, probiotics or dietary interventions, may also help stabilise the gut microbiota and prolong treatment benefits. The role of antibiotics pre-treatment to reduce baseline microbial load and enhance donor microbiota engraftment also warrants further investigation. Studies investigating the specific microbial changes associated with symptom relief could provide valuable insights into the potential mechanisms of action and underpin the development of targeted microbial therapies. Further exploration of donor selection criteria and investigation of the impact of repeated treatments on gut microbial diversity and function are essential for refining FMT protocols.

### Conclusion

In this observational extension study, repeated on-demand FMT was safe and feasible and conferred marked symptom relief in individuals with type 1 diabetes and gastrointestinal neuropathy. We suggest that FMT is a promising treatment for this chronic and debilitating condition that has few alternative treatments.

## Data Availability

Anonymised data may be made available upon reasonable request in accordance with the General Data Protection Regulation (GDPR). Access to de-identified participant data will require a formal data-sharing agreement, and, where applicable, approval from the relevant ethics committee. All inquiries and proposals should be directed to the corresponding author.

## Abbreviations

AE: Adverse event
CTCAE: Common Terminology Criteria for Adverse Events
FMT: Faecal microbiota transplantation
GSRS-IBS: Gastrointestinal Symptom Rating Scale for Irritable Bowel Syndrome

## Authors’ relationships and activities

SMDB has received lecture honoraria from Tillotts. MWK received support from Pfizer Biopharmaceuticals Group to attend the United European Gastroenterology Week in Vienna, 2024. CLH has received honoraria for lectures from Tillotts, Merck and Johnson & Johnson. Additionally, CLH is a member of the Expert Working Group at the European Directorate for the Quality of Medicines & HealthCare (EDQM) of the European Council (unpaid). The remaining authors declare that there are no relationships or activities that might bias, or be perceived to bias, their work.
